# High-Resolution Electron Diffraction of Hydrated Protein Crystals at Room Temperature

**DOI:** 10.1021/acsnano.3c05378

**Published:** 2023-10-27

**Authors:** Sergi Plana-Ruiz, Alejandro Gómez-Pérez, Monika Budayova-Spano, Daniel L. Foley, Joaquim Portillo-Serra, Edgar Rauch, Evangelos Grivas, Dominique Housset, Partha Pratim Das, Mitra L. Taheri, Stavros Nicolopoulos, Wai Li Ling

**Affiliations:** † NanoMegas SRPL, Rue Emile Claus 49, Brussels 1050, Belgium; ‡ Servei de Recursos Científics i Tècnics, Universitat Rovira i Virgili, Tarragona 43007, Catalonia, Spain; § Université Grenoble Alpes, CEA, CNRS, IBS, F-38000 Grenoble, France; ∥ Department of Materials Science and Engineering, Johns Hopkins University, Baltimore, Maryland 21218, United States; ⊥ SIMAP, Grenoble INP, Université Grenoble Alpes, CNRS, F-38000 Grenoble, France

**Keywords:** electron diffraction, liquid phase electron microscopy, protein electron crystallography, lysozyme, graphene liquid cell

## Abstract

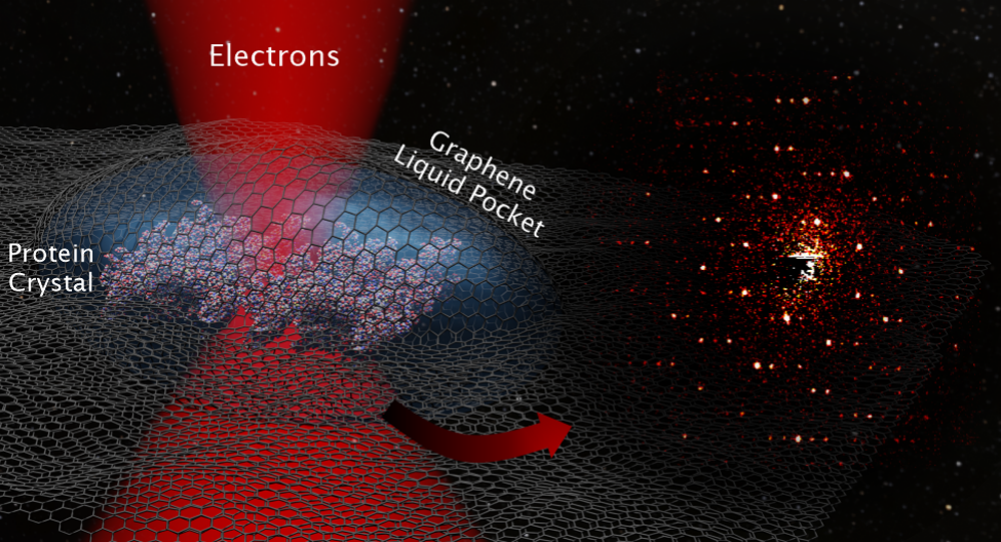

Structural characterization is crucial to understanding protein function. Compared with X-ray diffraction methods, electron crystallography can be performed on nanometer-sized crystals and can provide additional information from the resulting Coulomb potential map. Whereas electron crystallography has successfully resolved three-dimensional structures of vitrified protein crystals, its widespread use as a structural biology tool has been limited. One main reason is the fragility of such crystals. Protein crystals can be easily damaged by mechanical stress, change in temperature, or buffer conditions as well as by electron irradiation. This work demonstrates a methodology to preserve these nanocrystals in their natural environment at room temperature for electron diffraction experiments as an alternative to existing cryogenic techniques. Lysozyme crystals in their crystallization solution are hermetically sealed *via* graphene-coated grids, and their radiation damage is minimized by employing a low-dose data collection strategy in combination with a hybrid-pixel direct electron detector. Diffraction patterns with reflections of up to 3 Å are obtained and successfully indexed using a template-matching algorithm. These results demonstrate the feasibility of *in situ* protein electron diffraction. The method described will also be applicable to structural studies of hydrated nanocrystals important in many research and technological developments.

Crystallography is the most widely used method to determine three-dimensional (3D) structures of macromolecules such as proteins when suitable crystals are available. For X-ray crystallography, even when using nanometer-sized beams in synchrotron facilities, micrometer-sized crystals are typically required for successful structure determination. This constraint is particularly disadvantageous for protein crystals, which are difficult to form due to their weak intermolecular interactions. To be able to use submicrometer-sized crystals, serial crystallography in X-ray free electron lasers (XFELs) appears as one solution. These large facilities provide bright femtosecond X-ray pulses that allow diffraction patterns to be collected on individual nano- or microcrystals and further enable the monitoring of dynamics in protein complexes.^1^ However, few XFEL facilities exist in the world and the technique requires a very high crystal density (∼10^10^ crystals/mL). On the other hand, electron crystallography, another possible solution, uses commonly available transmission electron microscopes (TEMs) and requires only a small sample volume of suitable crystals for successful structure determination.

Structural models of small molecules and proteins have been determined by electron crystallography, generally referred to as 3D electron diffraction (3D ED) or MicroED.^2−5^ The common data collection strategy involves sample rotation similar to that employed in single-crystal X-ray diffraction.^3^ Serial electron crystallography, similar to serial X-ray crystallography at synchrotrons or XFEL facilities, has also been successfully applied to solve protein structures.^4^ Furthermore, charge information, often important in protein functions, has been elucidated in the electrostatic potential maps obtained from electron diffraction (ED) data.^5^ Despite these promising results, the number of protein structures solved by electron crystallography is still far behind those by X-ray crystallography or by single particle analysis with cryogenic electron microscopy (cryoEM), according to the statistics of the Protein Data Bank (PDB)^6^ and the Electron Microscopy Data Bank.^7^

A major difficulty lies in the preservation of the integrity of protein nanocrystals in ED experiments. All reported protein structures determined by ED to date are obtained from vitrified crystals. Vitrification preserves biological samples in their native hydrated state and enables them to be studied by cryoEM in a solid state compatible with the vacuum of the electron microscope column.^8^ For small protein samples, vitrification usually proceeds by the application of a small sample volume (2–4 μL) onto a holey carbon TEM grid, followed by blotting of excess sample solution with a filter paper and then plunge-freezing in liquid ethane.^9^ Although this methodology proves to be very successful, changes in the buffer conditions or the mechanical shock may undermine the crystal packing. Importantly, near liquid nitrogen temperature is far from the functioning temperature of proteins in biological conditions.

Liquid phase electron microscopy (LPEM) is an alternative to cryoEM to be explored for studying native protein nanocrystals. Liquid samples have been successfully isolated from the vacuum of the TEM using sophisticated liquid cells in the form of microfluidic chips fitted in specially designed sample holders.^10^ Such complex systems have given insights into inorganic^11,12^ as well as soft or biological samples.^13,14^ Although they are highly versatile, their image resolution is ultimately limited by the scattering along the combined thickness of the liquid and the viewing windows, which typically adds up to several hundred nanometers.^15^

For high-resolution LPEM, graphene liquid cells (GLCs) have been used. Graphene is a strong and flexible 2D material with high thermal and electrical conductivities, which are ideal properties for membrane supports in TEM grids. Graphene sheets have been used to encapsulate femtoliter sample volumes in liquid pockets to monitor nanoparticle nucleation dynamics as well as to visualize soft matter with atomic resolution.^16,17^ Besides being highly electron transparent, graphene acts as a radical scavenger and thus offers protection to the samples against radiation damage induced by electrons or X-ray.^18,19^ Whereas GLCs have yielded atomic resolution in TEM (and scanning TEM) imaging, very few works have been published on the benefits of such technology for ED experiments.^20−22^

The present work shows how GLCs can be used to encapsulate lysozyme crystals in their mother liquor so that high-resolution ED patterns of these crystals can be acquired at room temperature in their quasi-natural environment using standard TEM sample holders. These results would be applicable to 3D structural resolution of protein crystals or other hydrates in their liquid environment by electron crystallography.

## Results and Discussion

### Preparation of Liquid Cell with Lysozyme Nanocrystals

A key point to successfully acquire exploitable ED patterns from hydrated protein crystals lies in obtaining thin liquid pockets. To achieve the best signal-to-noise ratio, electron scattering from each component of the liquid cell needs to be carefully considered, as it determines the distribution of the diffracted intensity in the collected diffraction pattern. Specifically, the electron beam will interact with the top sealing membrane, followed by the liquid, the protein crystal, the liquid on the other side of the crystal, and finally, the bottom sealing membrane. While the liquid volume needs to be adequate to avoid protein denaturation, it needs to be minimized so that the diffraction signal will not be dominated by solvent scattering (background noise).^15^ In typical microelectromechanical systems (MEMS) microfluidic cells, silicon nitride membranes of 10–50 nm are used as sealing membranes and spacers of ∼100 nm or larger are used between the two membranes to define the liquid volume inside the system. On top of this nominal combined thickness, bulging due to the vacuum in the TEM can considerably increase the effective thickness of the cell and limit the final spatial and diffraction resolution.^23^

Compared with silicon nitride cells, GLCs can considerably shorten the path length across the sample assembly and reduce the inelastic scattering of the electron beam, which leads to better resolutions in both imaging and diffraction.^16,24^ Besides being much thinner than silicon nitride (graphene can be as thin as one monolayer, ∼0.35 nm), the flexibility of graphene membranes allows them to adapt to different particle dimensions and minimize liquid volumes. Membrane bulging is also reduced due to the high Young’s modulus of graphene. Furthermore, as graphene acts as a radical scavenger when the electron beam interacts with the liquid, the maximum electron dose (e-dose) that particles can sustain before losing their 3D order is higher. A higher e-dose can thus be used to achieve a higher signal-to-noise ratio during data collection.^17,18,25^

Another consideration in LPEM sample preparation is the size and shape of the sample. Introducing particles with an extended dimension, such as platelets or needles, into a prefabricated liquid cell can be challenging or even impossible. In this respect, GLCs present an additional advantage, as the graphene assembly can accommodate different sample morphologies.

Successful LPEM experiments also depend on well-sealed hermetic liquid cells. Ideally, the use of monolayer graphene as sealing membranes would be optimal, as it minimizes the background signal from the scattering in the membranes. Nonetheless, small cracks tend to appear in GLC with monolayer graphene, which result in the evaporation of the buffer liquid and protein denaturation. Therefore, even though it is certainly possible to properly seal protein crystals by means of monolayer graphene, GLCs made of TEM grids covered with 3–5 monolayers of graphene (1–1.7 nm) yield more robust liquid pockets and minimize the degradation of graphene produced by knock-on damage. Homemade^26^ and commercial graphene grids have been tested in this work. Preparing graphene grids can be time-consuming, and it is not trivial to achieve graphene surface free of (e.g. PMMA) residues. On the other hand, the quality of commercial grids has also been found to be inconsistent as some batches contain remnants of the copper substrate used to grow the graphene. Nevertheless, the remnants have not affected ED data collection owing to the large difference in unit cell parameters between protein crystals and copper nanoparticles. No observable difference in their ability to preserve hydrated protein crystals was detected between the homemade grids and commercial grids.

Electron microscopy grids covered with ultrathin amorphous carbon films of 2–3 nm in thickness have also been tested. In this case, micrometer-sized liquid pockets were more likely to form (see Figure S1), which led to diffraction patterns with a higher diffused background arising from the electron scattering in the larger liquid volume. Furthermore, the 3D order of the particles degraded more rapidly than in GLCs because amorphous carbon did not provide the same protection against radiation damage as graphene.

Lysozyme crystals, the benchmark for protein crystallography, were crystallized as nanocrystals for the liquid phase diffraction experiments described here. After crystallization optimization trials, bar-shaped lysozyme crystals were obtained with an acetate crystallization buffer (see Methods).^27−29^ The crystallization strategies generated unique populations of nanometer-sized crystals suitable for ED and at the same time easily identifiable in TEM images.

The lysozyme nanocrystals were also crystallized in D_2_O to explore its protective effect on macromolecules, which has been reported in the literature.^30^ Lysozyme solubility was significantly lower in D_2_O than in H_2_O due to the solvent isotope effect in the crystallization experiments. Similar observations have been made previously with other proteins, and it has been suggested that stronger attractive protein–protein interactions observed in D_2_O result from an enhanced hydrophobic effect in heavy water compared with light water.^31−33^

No significant difference was observed between the H_2_O and D_2_O crystals in the ED results except for an apparent higher degree of ordering, manifested as less intense diffused streaks among the ED reflections for the crystals in D_2_O (see Figure S2). As such streaking may also depend on the particular crystals examined and their orientations, a more in-depth and systematic study will have to be carried out in the future to provide a clear answer to the question of the impact of the isotopic effect (H_2_O/D_2_O) on radiation damage in protein crystals. (Results from H_2_O and D_2_O samples are not specifically differentiated below.)

The principle of liquid cell preparation is relatively simple. Around 200 nL of the sample solution containing the crystals in its mother liquor was deposited onto a graphene membrane supported by a holey carbon Cu TEM grid held by a pair of anticapillary reverse tweezers. Subsequently, a second grid with the graphene layer facing the drop was placed on top of the first grid. To facilitate the contact between the two graphene layers, 1/8 of the area at the edge of the top grid was cut as suggested by Hauwiller et al.^34^ Liquid sample was trapped in pockets as π–π stacking formed between the two graphene membranes when they came into contact.

**Figure 1:**
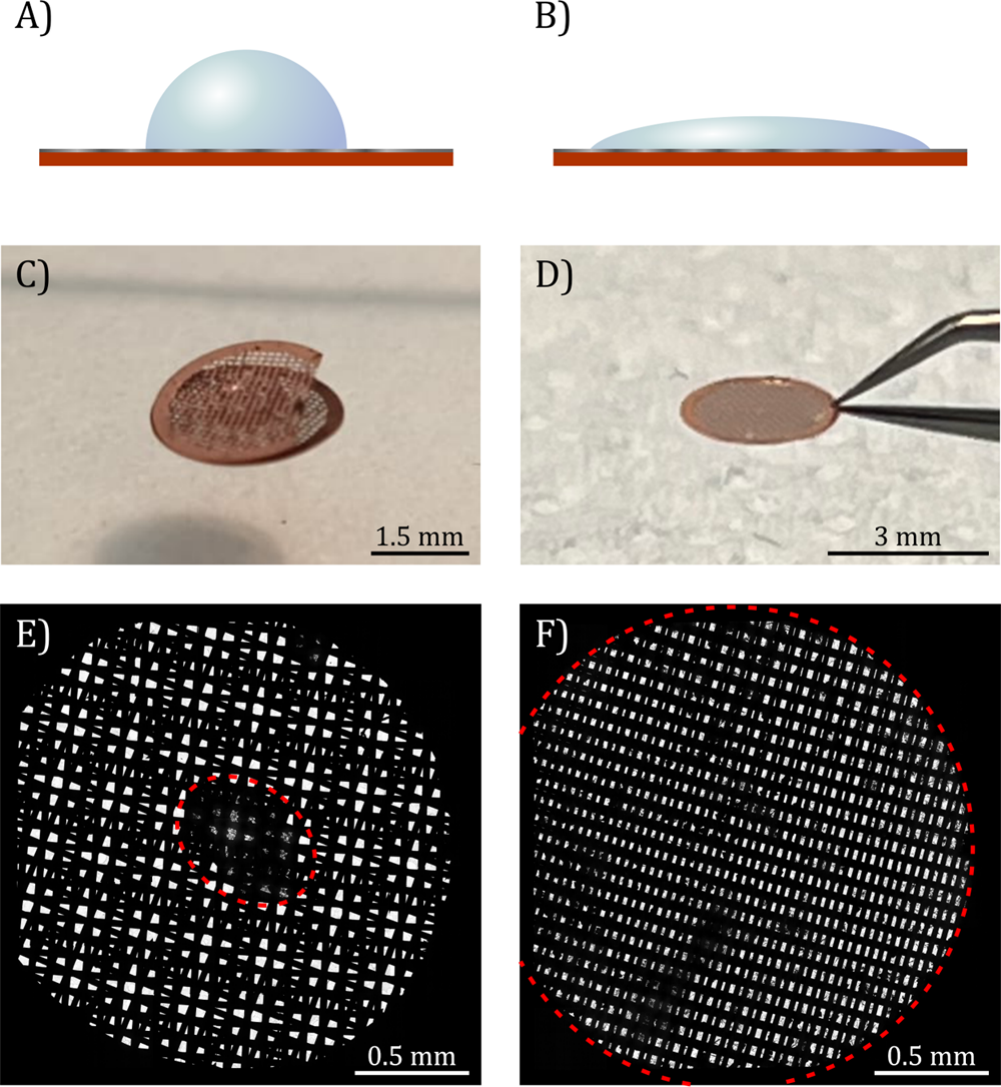
Liquid cell preparation with untreated and glow-discharged graphene membranes. (A, B) Schematic diagrams of a liquid droplet deposited onto untreated (A) and glow-discharged (B) graphene membranes. (C, D) GLCs with untreated (C) and glow-discharged (D) graphene grids. (E, F) Overview TEM images of GLCs assembled with untreated (E) and glow-discharged (F) graphene grids. The red dashed curves mark the limit of the spread of the sample drop in the respective GLCs.

Although the GLC assembly is straightforward, the successful formation of thin liquid pockets of biological samples necessitates reducing the hydrophobicity of the graphene membranes. Hydrophobicity of graphene has been known to cause a heterogeneous distribution of proteins when used as a support film for cryoEM experiments.^35,36^ In fact, aqueous solutions deposited onto untreated graphene do not wet the film but stay as a droplet to minimize the surface energy (Figure 1A). Figure 1C shows the scenario of an attempt to prepare a GLC using two untreated graphene grids. The droplet prevents the graphene sheets from getting into full contact for bond formation, and the use of a smaller volume will not improve the situation. As shown in Figure 1E, a small but round droplet is localized on the GLC, which is too thick for the electron beam to traverse. Even though thinner regions could be found at the edge of the droplet, most of the protein crystals in these regions were surrounded by salt crystals. The thickness of the droplet had prevented the tight sealing of the two graphene sheets around the droplet, which had led to buffer evaporation, salt crystallization, and denaturation of the protein crystals. (This observation also emphasized the importance of minimizing the time taken to assemble the liquid cell and of working in a high humidity environment.)

To render the graphene layers hydrophilic, a mild glow discharge was applied to the graphene coated grids used to assemble the GLCs. Glow discharge functionalizes graphene with O and OH groups.^37^ This surface modification partially transforms graphene into graphene oxide, which has a lower thermal and electrical conductivity^38^ and a lower performance as a radical scavenger. Although this treatment partly reduces the protective effect of the graphene layers against beam damage, the short glow discharge (∼30 s) greatly enhances the wettability of the membrane and allows the effective formation of liquid pockets. Indeed, when glow-discharged graphene grids were used, the two grids were found to snap into contact when the top grid was placed above the grid with the spread sample. As Figure 1F testifies, a more homogeneous distribution of liquid sample across the GLC is obtained in this case.

### Electron Diffraction Data Collection Strategy

**Scheme 1:**
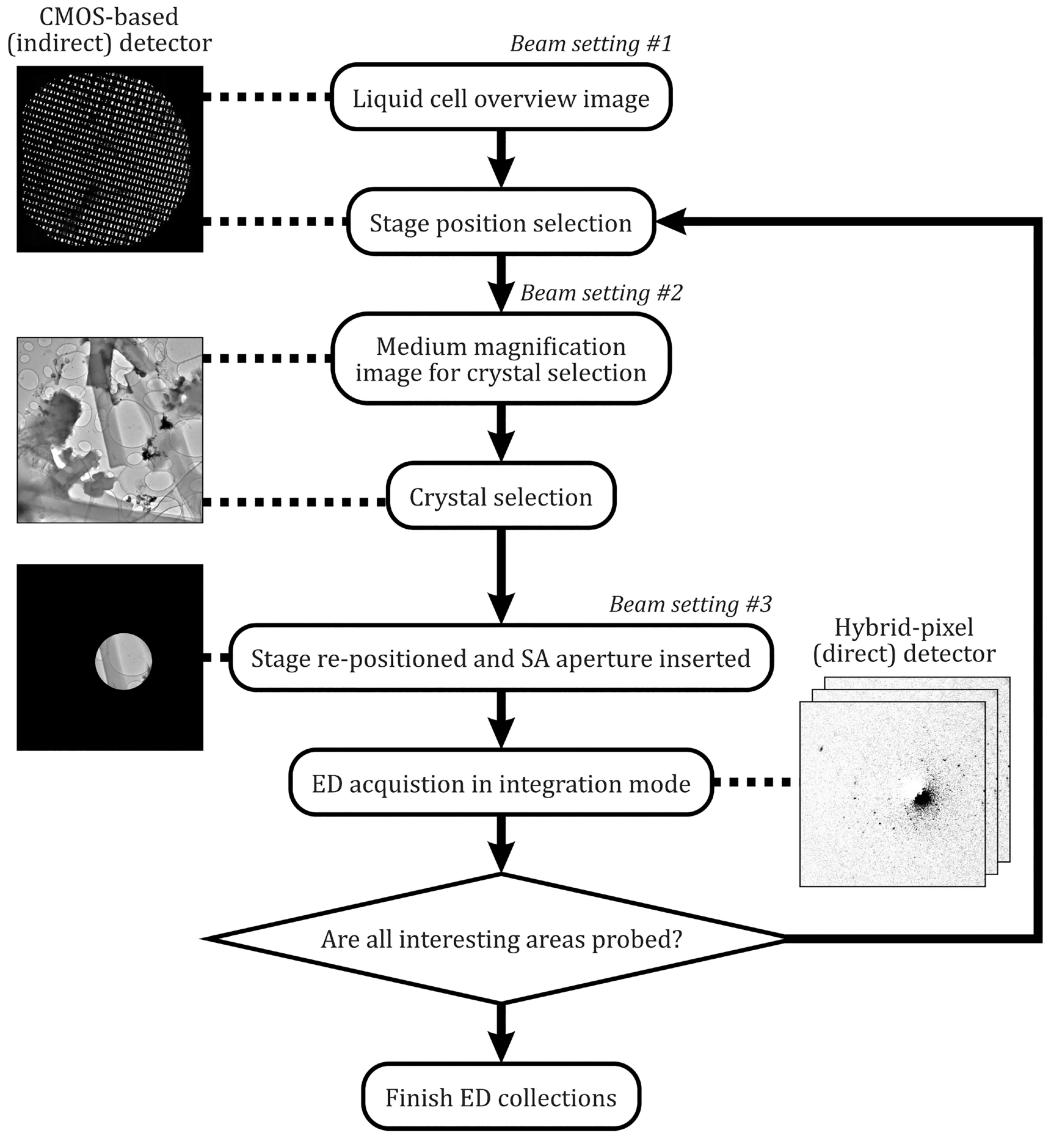
Data Collection Strategy for the Acquisition of ED Patterns from Liquid Pockets Found across the GLC Search for suitable crystals is performed on a scintillator-based CMOS detector, and ED patterns are collected on a hybrid-pixel direct electron detector.

After liquid cells were prepared according to the methodology explained in the previous section, they were loaded onto a standard single-tilt TEM holder for imaging and diffraction. Scheme 1 shows the routine established in this work for the acquisition of ED patterns from areas selected across the GLC.

In this scheme (Scheme 1), two different cameras are used to collect images and diffraction patterns, respectively. Images are collected on a 4k × 4k complementary metal oxide semiconductor (CMOS)-based fiber optic coupled detector with a small physical pixel size. This camera enables the acquisition of high-resolution images with a large field-of-view. With this camera, features in the specimen can be discerned easily and potentially well diffracting crystals can be efficiently identified. On the other hand, optical coupling introduces noise, which reduces the detector signal-to-noise ratio and limits its ability to record weak reflections in protein diffraction patterns. Instead, a hybrid-pixel direct electron detector is used to capture the ED patterns. The larger physical pixel size and the thicker semiconductor sensor of this technology allow data acquisition with intense and/or very weak electron beams with virtually zero noise. The Medipix3 sensors used in this detector provide reliable counts without coincidence loss at low exposure times even with very weak electron beams (i.e. good linearity between incoming number of electrons and detected counts).^39^ The combination of these two detectors gives satisfactory results in this experimental setup, as it takes advantage of the best features of the two technologies that have been specifically designed for TEM imaging and diffraction, respectively.

The data collection routine used in the experiments is very similar to that used in cryoEM experiments except for the fact that ED patterns instead of images are acquired in the final step. The electron beam is deflected away from the sample (beam blanked) and illuminates the liquid cell only when one of the two detectors is activated. Importantly, crystals of interest are exposed to a negligible dose during search or focus and a significant e-dose only during the diffraction data acquisition.

The workflow consists of three different predefined beam settings, which are registered in the data collection software. A first low beam current and low magnification setting (e.g., high spot size and fully spread beam at 120× magnification) are used to acquire an overview (atlas) of the whole liquid cell to look for suitable electron transparent areas populated by crystallites. This atlas is composed of 49 (7 × 7) images stitched together according to their stage position (e.g., Figure 1E,F). A second low beam current and medium magnification setting (e.g., high spot size and slightly condensed beam at 1700× magnification) are used to identify possible crystal targets. Finally, a third higher beam current setting (e.g., low spot size and well spread beam) with a selected area (SA) aperture is used for ED data acquisition (at 1–1.2 m of nominal camera length). During the initial setup of the TEM configurations, the SA aperture is centered in the imaging mode with the third beam setting at 5000× magnification. This aperture is inserted manually whenever a good crystal is located, as the microscope is not equipped with a motorized insertion system. Nevertheless, the aperture position is found to be stable with repeated insertions and removals. The stage positions and imaging areas of the three beam settings are carefully aligned with each other to ensure that diffraction patterns are acquired at the correct location of the crystals selected for data collection.

As shown in Scheme 1, the acquisition protocol unfolds as follows. After an atlas of the GLC is obtained, a region with potentially good (thin and isolated) crystals is identified and the stage is moved to that region accordingly. An image of the region is then acquired with the second setting to identify a good crystal target. Once the target is selected, the sample is not exposed again until diffraction data acquisition. Using the alignment parameters registered in the data acquisition software, the stage is moved to a location such that the target is centered in the area defined by the SA aperture in the first image plane that contributes to the diffraction pattern. The SA aperture is then inserted, the third configuration is set, and the microscope is switched to the diffraction mode for ED data acquisition. In this way, the e-dose that the crystal is subjected to comes almost solely from the ED acquisition.

### Evaluation of Crystal Integrity

**Figure 2:**
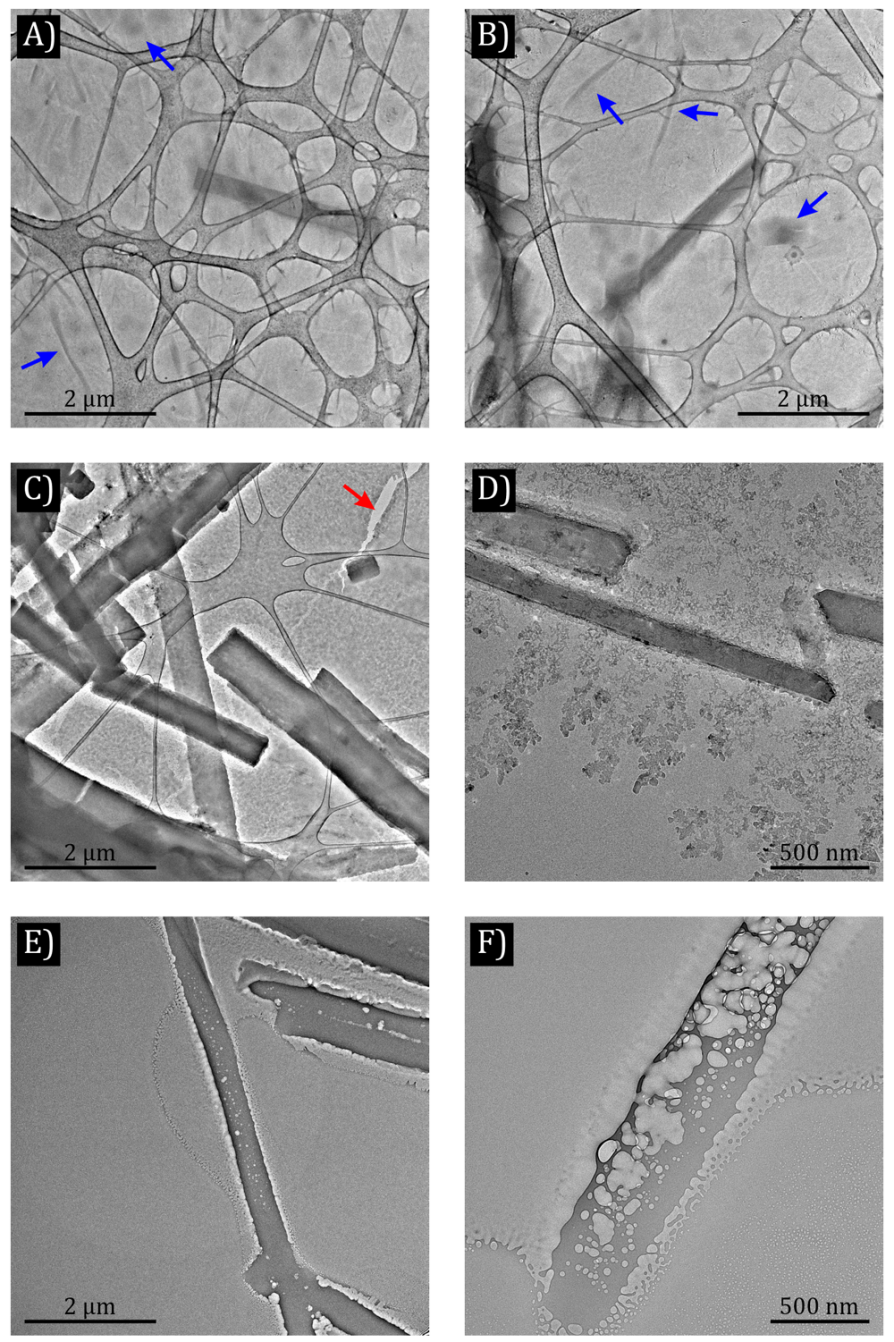
TEM images of lysozyme crystals in liquid cells. (A, B) Crystals in properly sealed liquid pockets. Blue arrows point to examples of localized liquid pockets formed by folds in the graphene membranes. (C) Crystals with compromised crystallinity near a cracked graphene membrane (red arrow). (D) Dendritic salt crystals nucleated around the lysozyme crystals as the buffer solution dried out during a flawed GLC preparation. (E, F) Lysozyme crystals in well-sealed liquid pockets between amorphous C membranes undergoing dissolution under an intense electron beam.

Whereas it is easy to identify the bar-shaped lysozyme crystals in the imaging mode, it is not easy to directly evaluate their diffracting power. The ability to foretell the crystallinity of the sample particles is key to efficient data collection. Obviously, areas with cracked graphene layers (e.g., Figure 2C) are to be avoided as the sealing of the liquid pockets in these regions is likely compromised. The presence of buffer liquid is often indicated by a diffused contrast gradient around the particles. Figure 2A,B shows examples of TEM images with protein crystals encapsulated in liquid pockets of a GLC. Small and thin bar-shaped crystals with well-defined straight edges are found in both images. Folds in the graphene layers forming localized liquid pockets of the mother liquor (blue arrows) can also be observed in the field-of-view. On the other hand, irregular edges along the bars often indicate the nucleation and growth of salt crystals, which is a sign of liquid loss through evaporation (Figure 2C). Dendritic salt crystals can also form in severe cases, as exemplified in Figure 2D. Even though the dehydrated particles retain their crystal shape, no reflections have been detected in their diffraction patterns.

For comparison, amorphous carbon films were also employed for the liquid cell assembly. Significantly larger liquid pockets were obtained in this case (see Figure S1). Although the presence of a larger liquid volume is more favorable for preserving the crystallinity of the protein crystals, it is not optimal for ED experiments for two reasons. The first reason is that the extra liquid leads to diffuse scattering. The scattering contributes to a prominent background in the ED pattern, which masks the weak protein reflections. The second reason is radiolysis of the extra buffer solution, which accelerates the dissolution of the protein crystal upon electron beam irradiation (see Figure 2E,F). For these reasons, carbon film liquid cells have not provided any diffraction patterns with quality comparable to those from GLCs.

**Figure 3:**
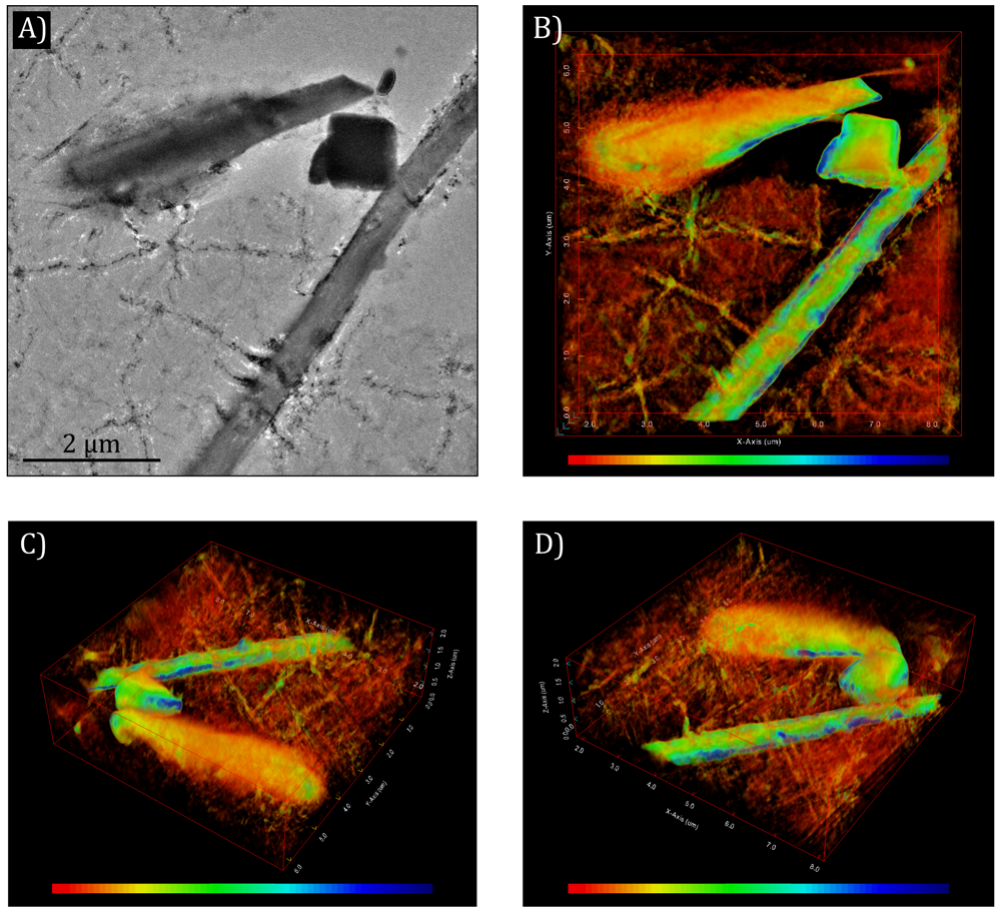
Electron tomography of two lysozyme crystals in a liquid cell with amorphous carbon membranes. (A) TEM image in the tilt series. (B–D) Different projections of the reconstructed 3D volume. Diffused contrast indicating the presence of a liquid pocket is observed around one end of the bar-shaped crystal while the other end of the crystal seems dry. The cube-like particle is likely a salt (NaCl) crystal from the buffer solution. Dendrites can also be observed in the reconstructed volume. The color bar represents the scale of electron density in the rendered volume.

After the evaluation of more than 200 lysozyme crystals in GLCs, some lysozyme particles were found to diffract even in the absence of the surrounding diffused contrast that indicated the presence of liquid. In other words, large buffer liquid pockets were not strictly necessary to preserve lysozyme crystals; a thin liquid layer that maintained the humid environment was sufficient. In fact, the formation of large liquid pockets in graphene sandwich is uncommon and pockets of more than 1 μm in diameter are rare.^40^ Considering the size of the bar-shaped particles (few μm in length, up to 1.5 μm in width, and less than 1 μm in thickness), particles were more often found to be only partially immersed in liquid (manifested as diffused contrast). Figure 3 shows results from an electron tomography carried out in the image mode with a liquid cell assembled with amorphous carbon membranes, in which a liquid pocket is retained at one end of the particle while the rest seems dry, as indicated by the surrounding dendritic salt crystals. The rendered 3D volume allows for the thickness determination of the surrounding buffer solution, which ranges from 200 to 400 nm on the wet part of this rather thick crystal (see Figure S3). In this context, the crystal integrity could not be evaluated by imaging but solely by diffraction.

### Electron Diffraction

Diffraction patterns from targeted particles were acquired with e-doses between ∼0.01 e^–^/(Å^2^ s) and ∼0.03 e^–^/(Å^2^ s). Since the effect of radiation exposure on a crystal depends on many factors (crystal thickness, volume of surrounding liquid, etc.), it is difficult to predict the maximum e-dose that a crystal can tolerate.^41^ To circumvent the *a priori* unknown dose limit, the integration mode of the hybrid-pixel detector was used in the data collection (see Methods section). This mode enabled the continuous collection of 3000 (still) frames in total for each crystal with an exposure time of 10 ms per frame (30 s total exposure, ∼0.3 e^–^/Å^2^ to 1 e^–^/Å^2^ cumulative e-dose).^4^ In this way, the damage from the electron beam could be evaluated after the acquisition by following the evolution and eventual decay of the reflection intensities throughout the frame sequence. The frames before a significant decrease in the signal-to-noise ratio were then used to obtain the final ED pattern for further analysis.

**Figure 4:**
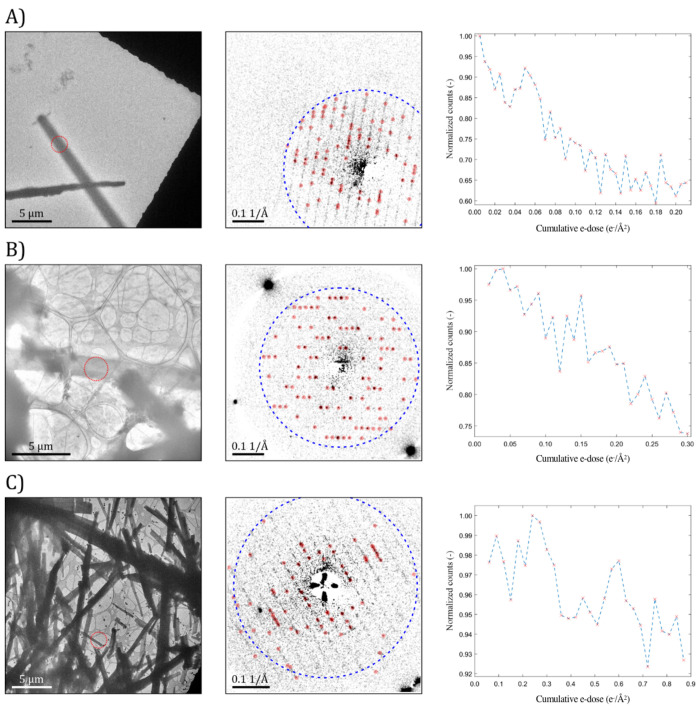
Comparison of radiation resistance of lysozyme crystals in three different liquid cell scenarios by continuous sampling of the diffraction patterns. (A) A thick crystal encapsulated between amorphous carbon membranes, (B) a thick crystal in a GLC prepared with 3–5 graphene layers, and (C) a thin crystal in a GLC prepared with 3–5 graphene layers. The red dashed circles in the TEM images represent the area contributing to the diffraction, as defined by the SA aperture. The red filled circles in the background-subtracted ED patterns indicate the positions where reflection intensities are detected throughout the acquired ED series (3000 frames in total; 30 s total exposure). The intensities at these positions are summed to obtain the total single-crystal diffraction intensity shown in the plots. Each data point corresponds to the integrated intensity from 100 consecutive frames in the series. Reflection intensities are considered in the resolution rings between 16.8 and 4.0 Å in (A), 15.5 and 3.9 Å in (B), and 15.4 and 3.8 Å in (C). The blue-dashed circles in the ED patterns mark the 4 Å resolution ring. The results show that crystals in GLC with 3–5 layers of graphene are more resistant to radiation damage than crystals wrapped in amorphous carbon. Thinner samples also sustain less radiation damage than thicker samples.

Although the standard e-dose limits generally acceptable for ED and single particle analysis in cryoEM are ∼2 e^–^/Å^2^ and 30 e^–^/Å^2^, respectively,^42^ beam damage is noticeable below 0.1 e^–^/Å^2^ at room temperature. Figure 4 shows representative lysozyme crystals in three different conditions. Figure 4A corresponds to the result of a thick crystal encapsulated in amorphous C membranes. The intensity plot indicates that the single-crystalline signal falls sharply by ∼30% after 0.1 e^–^/Å^2^ of cumulative e-dose. Figure 4B shows another case of a thick crystal but encapsulated in a GLC. The crystallinity is significantly better preserved in comparison to the amorphous carbon cell, yet degradation is still obvious at around 0.2 e^–^/Å^2^. Figure 4C represents the optimal scenario with a thin and isolated crystal encapsulated in a GLC. In this case, the single-crystalline intensity decreases by only ∼10% with 0.9 e^−^/Å^2^ cumulative e-dose, as illustrated in Figure 4C (see Figure S2 for more examples).

Background subtraction was performed on the ED patterns displayed in Figure 4. For each acquisition, strong high-resolution reflections were monitored through the frame series to determine the number of frames to be integrated for a good signal-to-noise ratio without losing the weak reflections. Nonetheless, the background was often still high in the final integrated diffraction pattern. This background signal originated from the different components of the liquid cell, e.g., the amorphous carbon (carbon membrane or lacey carbon support for graphene layers), the buffer solution, and any amorphous part of the protein particles, which possibly resulted from radiation damage. To eliminate such strong contributions and to enhance the reflection signals, a 2D averaged radial profile centered around the primary beam of each pattern was generated, excluding the area around the detected reflections. The resulting mask was then applied to the integrated pattern. Compared with the raw data, reflections in the background subtracted images were considerably easier to detect (see Figure S4 for details and examples).

**Figure 5:**
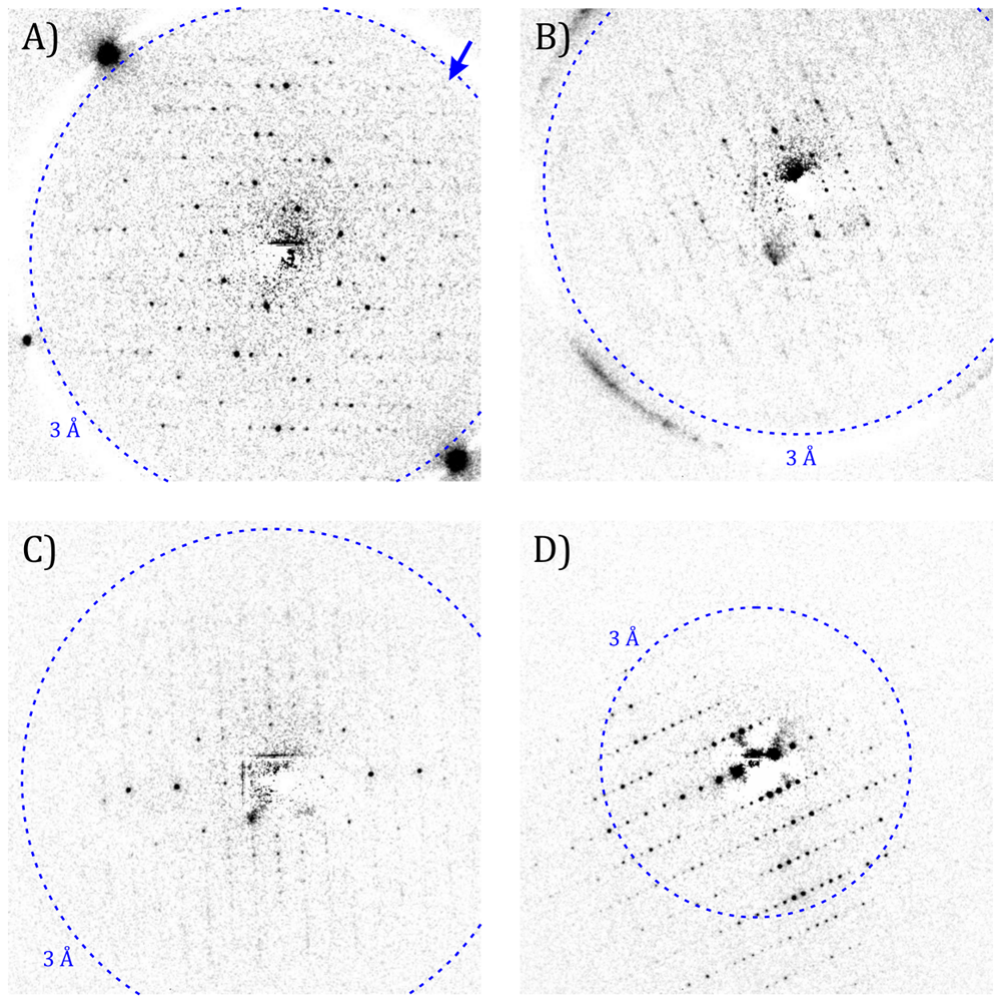
Electron diffraction patterns of lysozyme crystals preserved in different conditions. (A) At room temperature in GLC with 3–5 graphene layers (0.15 e^–^/Å^2^ cumulative e-dose). The blue arrow points to reflections at ∼3 Å resolution. (B) At room temperature in GLC with monolayer graphene layers (0.05 e^–^/Å^2^ cumulative e-dose). (C) At room temperature in liquid cells with amorphous carbon layers (0.06 e^–^/Å^2^ cumulative e-dose). (D) At near liquid nitrogen temperature in amorphous ice vitrified by plunge freezing in liquid ethane on an amorphous holey C TEM grid (0.3 e^–^/Å^2^ cumulative e-dose). Reflections of up to 2.2 Å resolution can be observed.

Background subtracted diffraction patterns of lysozyme crystals in liquid cells at room temperature are compared with that of a vitrified crystal acquired using cryoEM in Figure 5. As shown in Figure 5A–C, reflections in the ED patterns obtained from crystals in the GLCs extend up to 3 Å resolution. In comparison, those from vitrified crystals of the same batch recorded close to the liquid nitrogen temperature reach 2.2 Å resolution (see Figure 5D). Reflections from the graphene layers do not appear here as their first reflections are located at 2.13 Å ({11̅00} family of planes), which is beyond the detector range at the camera lengths used in these experiments. Whereas liquid cells are free from ice crystal contaminations found in vitrified samples, strong reflections and diffraction rings can be generated from salt crystals due to the evaporation of the mother liquor in liquid cell samples (Figure 5A,B). In addition, Figure 5B,C displays some diffused streaks between Bragg reflections, which are not observed in cryogenic conditions. The streaking may indicate that correlated intermolecular displacements between molecular envelopes or conformational ensembles with short-range periodicities are enhanced at room temperature. (see Figures S5 and S6 for more images and ED patterns in H_2_O and D_2_O buffer solutions, respectively.)

**Figure 6:**
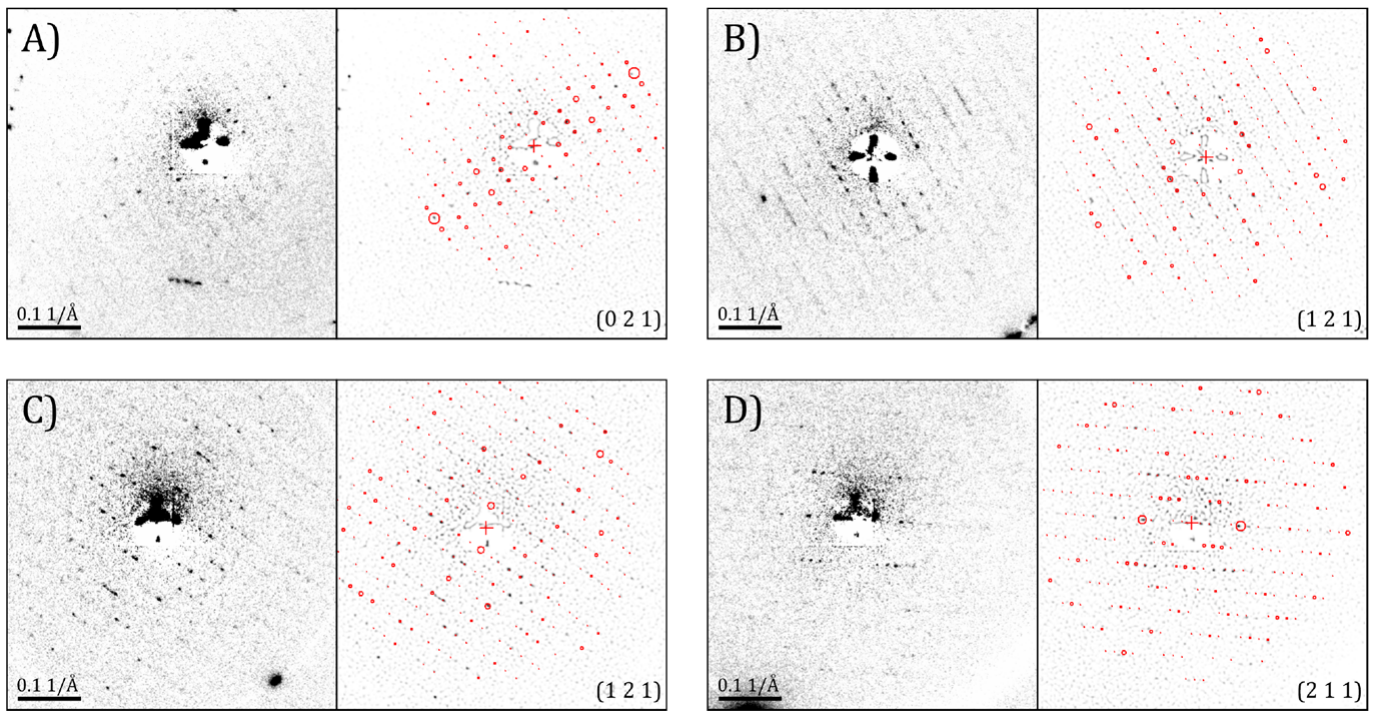
Electron diffraction patterns of lysozyme crystals encapsulated in a GLC (cumulative e-dose of ∼1 e^–^/Å^2^) and indexing results from the template-matching algorithm implemented in ASTAR.^43^ The simulated ED patterns with the reflections circled in red correspond to the best fit to the experimental data. (A) and (B) are crystals in H_2_O buffer liquid, and (C) and (D) are crystals in D_2_O.

The ED patterns were indexed *via* a template-matching algorithm based on the simulated ED patterns obtained from a kinematical calculation of a reported structure in the orthorhombic form (31.472 Å, 92.350 Å, 114.239 Å; *P*2_1_2_1_2; PDB code 4DC4(27)). The possibility that the assumed crystal structure does not correspond exactly to that in the experiment cannot be excluded. Such a scenario would lead to discrepancies between the simulated reflection intensities and the measured intensities, even though such discrepancies could also arise from dynamical effects and from the fact that the intensities recorded here in the still frames are only partial intensities. Nonetheless, the algorithm has been sufficiently robust and has effectively found the same zone-axis for ED patterns that appear geometrically similar to each other. Figure 6 shows four different ED patterns and their best matched indexing. A preferred orientation near the (0 2 1) direction is observed. The tendency to obtain the same zone axis in ED experiments, similar to texturing in powder X-ray diffraction, is highly dependent on the shape of the crystal. In this case, the bar-shaped particles tend to lie flat on the graphene membrane with the *a*-axis in the plane of the GLC assembly close to the perpendicular direction of the electron beam. Thus, most of the crystals yielded similar ED patterns. Different crystal orientations can be obtained by tilting the sample holder, as exemplified by Figure 6D, where the sample was tilted to 20°.

## Conclusions

The LPEM results presented in this work show that protein crystallography in a quasi-natural environment is possible at the nanoscale using conventional TEMs and standard sample holders. Apart from careful management of the electron beam illumination by low-dose imaging, the data collection strategy presented here does not require any specific equipment other than a sensitive detector with a high dynamic range to acquire statistically significant intensities for the very weak protein reflections. Liquid cells sealed with 3–5 graphene layers are shown to be optimal for GLC assemblies. These assemblies have satisfactory performance in the reduction of electron beam damage as well as in the preservation of hydrated protein crystals. The measurement conditions are described as quasi-natural because the liquid cells are still under vacuum in the TEM. However, liquid state at room temperature would be closer to the physiological environment compared with solid (vitrified) state at near liquid nitrogen temperature in cryoEM. Moreover, LPEM avoids the air–water interface, which tends to cause protein denaturation.^44^

Despite the apparent inferior LPEM data quality compared to data collected from vitrified crystals, the attained resolution of the hydrated lysozyme crystals encapsulated in GLCs is sufficient to obtain successful indexing of the ED patterns. Crystal structure determination by serial electron crystallography analysis should be possible once enough reflections are obtained.^4,45^ However, several hundreds of electron diffraction patterns will be necessary. Acquiring such data volume would require automatic data acquisition schemes similar to those developed for cryoEM single-particle analysis but in diffraction mode.^46^ Ideally, scanning TEM with a small diameter beam should be employed to limit the area of radiation damage during exposure.^4^ Precession ED can also be considered to obtain a higher number of symmetry-related reflections for more reliable indexing^47,48^ (see Figure S7).

Perhaps not surprisingly, diffuse scattering in lysozyme crystals, which was not present at cryogenic conditions, was observed with crystals sealed in GLCs at room temperature. Room-temperature X-ray studies on protein crystals have demonstrated that diffuse scattering carries dynamical information of the molecules, which can reveal biochemically relevant vibrational modes important to protein functions.^49−51^ Nonetheless, crystal deformation (e.g., bending) or disorder induced by the electron beam may also contribute to the diffuse scattering background in the GLC experiments. Further experiments will be essential to separate effects coming from electron beam damage and effects originating from protein internal dynamics.

In conclusion, this work shows that protein crystals can be studied in their mother liquor at room temperature in a simple and reproducible manner. Nonetheless, little is known to date about the effect of the electron beam on protein crystals in liquid environments. Future studies for a better understanding of the physical processes involved would be necessary to fully exploit the potential of liquid phase ED. Systematic evaluation of the surface tension of the protein buffer solution may also be performed to increase the efficiency of liquid pocket formation. Besides, the use of D_2_O vs H_2_O against radiation damage calls for more in-depth studies. The present results would encourage future structural studies and exploration of crystallization dynamics with liquid phase electron crystallography, not only for protein crystals but also for many inorganic and organic hydrates important in diverse research fields ranging from air pollution and biomedicine to food and building industries.^52^

## Methods

### Crystallization Procedure for Lysozyme Nanocrystals

Hen egg-white lysozyme was purchased from Sigma-Aldrich as a lyophilized powder and dissolved in distilled or heavy water (Euriso-Top, 99.92% D_2_O) to obtain stock solutions with a final concentration of about 70 mg/mL. The protein concentration was measured *via* the UV absorbance at 280 nm.

The previously developed rational crystal growth strategies to select the initial crystallization mixtures were used.^28,29^ In a batch with a total volume of 50 mL, 25 mL of protein stock solution at a concentration of 70 mg/mL was mixed with appropriate amounts of salt and buffer stock solutions to obtain mixtures at the final target salt and buffer concentrations (1.0–1.4 M NaCl, in 100 mM Na acetate buffer pH(pD) = 4.5). The mixtures were made in the presence of either distilled water (light water) or heavy water.

Prior to dissolution/dilution, the proper amount of NaCl and volume of glacial acetic acid were dissolved/diluted in distilled water or heavy water to obtain stock solutions with concentrations (5 M NaCl in 1 M sodium acetate buffer, pH(pD) = 4.5). The pD was adjusted with NaOD (Euriso-Top, 99% D) according to the formula pD = pH_meas_ + 0.3314*n* + 0.0766*n*^2^, where *n* = %D_2_O.^53^ All of the solutions were filtered through 0.22 mm Millipore filters.

### Transmission Electron Microscopy Grids for Graphene Liquid Cell Assemblies

The commercial graphene TEM grids consisted of a single layer or 3–5 layers of chemical vapor deposited (CVD) graphene placed on a lacey carbon support film of a 300 mesh Cu grid. They were purchased from Electron Microscopy Sciences (EMS) with references 1GLC300Cu and 3GLC300Cu, respectively. The ultrathin continuous carbon films were also purchased from EMS with references CF150-Cu-UL for 150 mesh Cu grids and CF300-Cu-UL for 300 mesh Cu grids. Homemade graphene grids were obtained following the procedure described by Duong *et al*. but using bilayer graphene instead of monolayer graphene.^26^ Glow discharge was applied with the GloQube Plus system from Quorum with a negative current of 20–25 mA for 30 s in an ambient air atmosphere of 0.15 mbar.

### Transmission Electron Microscopy

A FEI Tecnai F20 microscope with a Schottky field emission gun operated at 200 kV was used for the data collection. Microprobe mode, gun lens 3 and a 100 μm condenser aperture were used to achieve a low intensity and quasi-parallel electron beam. The spot size was varied between 5 and 8, and Thermo Fisher Scientific (TFS) EPU software was used to store and recall the different illumination settings used during the data acquisition protocol. Diffraction patterns were collected with a 70 μm SA aperture that corresponds to a circular area in the image plane of 2 μm in diameter. Images were obtained with a TFS Ceta camera, a CMOS-based and optical fiber coupled detector with 4096 × 4096 pixels, and a physical pixel size of 14 μm. This camera was directly controlled by the TFS EPU software. Diffraction patterns were acquired with the Amsterdam Scientific Instruments (ASI) CheeTah M3 detector, which is a hybrid-pixel direct electron detector. This detector consists of four Medipix3 sensors of 256 × 256 pixels (physical pixel size of 55 μm) arranged in a square, resulting in output frames of 512 × 512 pixels. It was controlled by the Dexter software provided by ASI and triggered by the DigiStar P1000 scanning/precession unit from NanoMEGAS SPRL. This detector can operate in sequential mode with a dynamic range of 24 bits and frame rate up to 700 Hz or in continuous mode with 12 bits and up to 2 MHz. For the experiments in this work, a sequential mode at 100 Hz was used. The continuous fast sampling allows for monitoring of diffraction quality degradation. Frames before high-resolution information loss are integrated for ED data with an optimal signal-to-noise ratio in each diffraction data set.

### Vitrification and Cryoelectron Microscopy

A TFS Vitrobot Mark IV was used for vitrification of the lysozyme sample. Around 4 μL of the sample solution with lysozyme nanocrystals was applied onto a glow-discharged Quantifoil holey carbon TEM grid at 4 °C and 100% relative humidity. The grid was then plunged frozen in liquid ethane and mounted onto a Gatan 626 cryotransfer holder for cryoEM experiments.

### Electron Diffraction Data Analysis

Original Matlab scripts were written for preliminary processing and for applying a flat-field correction to the raw ED patterns obtained from the Dexter software (TIFF format; 32-bit unsigned integer without compression). Background subtraction was carried out with the Diffractem Python package through a 2D average radial profile mask.^54^ Reflection positions were automatically located by the peakfinder8 algorithm from the CrystFEL package^55^ as well as by a clustering method available in the eADT program.^56^ Indexing was performed using the template-matching algorithm implemented in the ASTAR commercial software (Nanomegas SPRL).^43^

### Image Tomography Reconstruction

The protein crystal was identified in a liquid cell assembled with ultrathin amorphous carbon membranes. 4k × 4k images were acquired (TFS Ceta camera) from α-angles ranging between −46° and 40° in a standard single-tilt holder with a tilt step of 2° (45 images in total). A magnification of 5000× was used in the TEM to obtain a reconstruction of the whole bar-shaped crystal (2.012 nm/pixel). Images were binned to 512 pixels × 512 pixels to relax the processing load of the reconstruction process. Although the resolution is reduced, the SNR is increased by averaging the neighboring pixels during binning. Images were then aligned using the TomoJ^57^ (v2.6) plugin for tomographic reconstruction in ImageJ^58^ (v1.53e) image processing program. Image brightness and contrast were normalized after the alignment. The final 3D volume was reconstructed using the total variation minimization reconstruction algorithm implemented^59^ in original Matlab scripts written for this work. The reconstructed volume was further cropped to 480 pixels × 480 pixels × 128 pixels^3^ and rendered through the Visualizer-evo (v1.3.17.0) program available in the TEMography commercial software (System in Frontier Inc.).

## Supporting Information Available

Additional TEM images, electron diffraction patterns, data processing examples for background subtraction, and electron dose analysis (PDF)
Video of the reconstructed volume of the image tomography rotated with respect to *x* axis of the volume (MP4)
Video of the reconstructed volume of the image tomography rotated with respect to *y* axis of the volume (MP4)

## Abbreviations

3D: three-dimensional
XFEL: X-ray free electron laser
TEM: transmission electron microscope
ED: electron diffraction
cryoEM: cryogenic electron microscopy
PDB: Protein Data Bank
LPEM: liquid phase electron microscopy
GLC: graphene liquid cell
MEMS: microelectromechanical systems
CMOS: complementary metal oxide semiconductor
CVD: chemical vapor deposition
