# Supplementary material for: High-Resolution Electron Diffraction of Hydrated Protein Crystals at Room Temperature
Source: ACS Nano. 2023 Oct 27;17(24):24802–13. doi: 10.1021/acsnano.3c05378 (PMC10753879; doi:10.1021/acsnano.3c05378)
Supplement: Supplementary file 1 — nn3c05378_si_001.pdf [file nn3c05378_si_001.pdf]

High-Resolution Electron Diffraction of Hydrated Protein Crystals at Room Temperature

Sergi Plana-Ruiz,[1,2] Alejandro Gómez-Pérez,[1] Monika Budayova-Spano,[3] Daniel L. Foley,[4] Joaquim Portillo-Serra,[1] Edgar Rauch,[5] Evangelos Grivas,[1] Partha Pratim Das,[1] Dominique Housset,[3] Mitra L. Taheri,[4] Stavros Nicolopoulos,[,1] Wai Li Ling[*,3]*

[1]NanoMegas SRPL, Rue Emile Claus 49, Brussels 1050, Belgium.

[2]Servei de Recursos Científics i Tècnics, Universitat Rovira i Virgili, Tarragona 43007, Catalonia.

[3] Université Grenoble Alpes, CEA, CNRS, IBS, F-38000 Grenoble, France.

[4]Department of Materials Science and Engineering, Johns Hopkins University, Baltimore, MD 21218 Baltimore, USA.

[5]SIMAP, Grenoble INP, Université Grenoble Alpes, CNRS, F-38000 Grenoble, France.

SUPPORTING INFORMATION

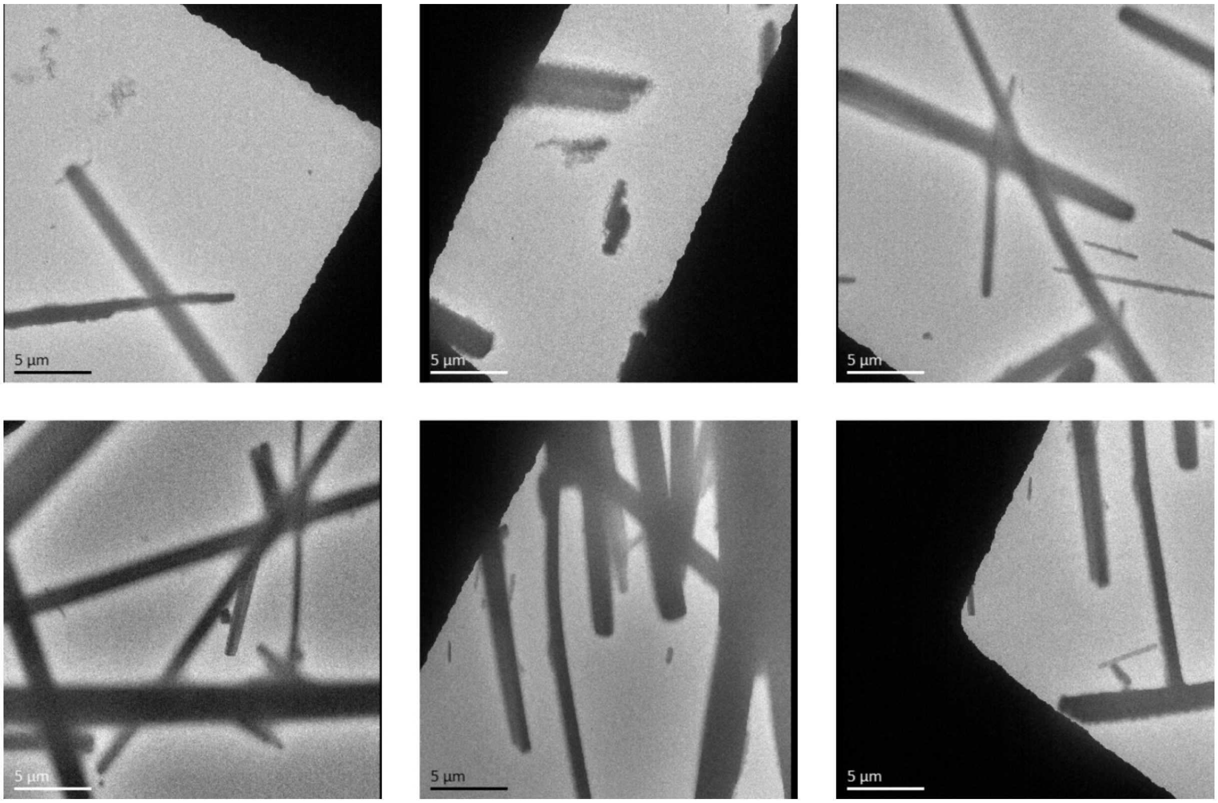

Figure S1. Transmission electron microscopy (TEM) images of lysozyme crystals encapsulated in liquid cells formed by two TEM grids coated with ultra-thin continuous amorphous carbon film.

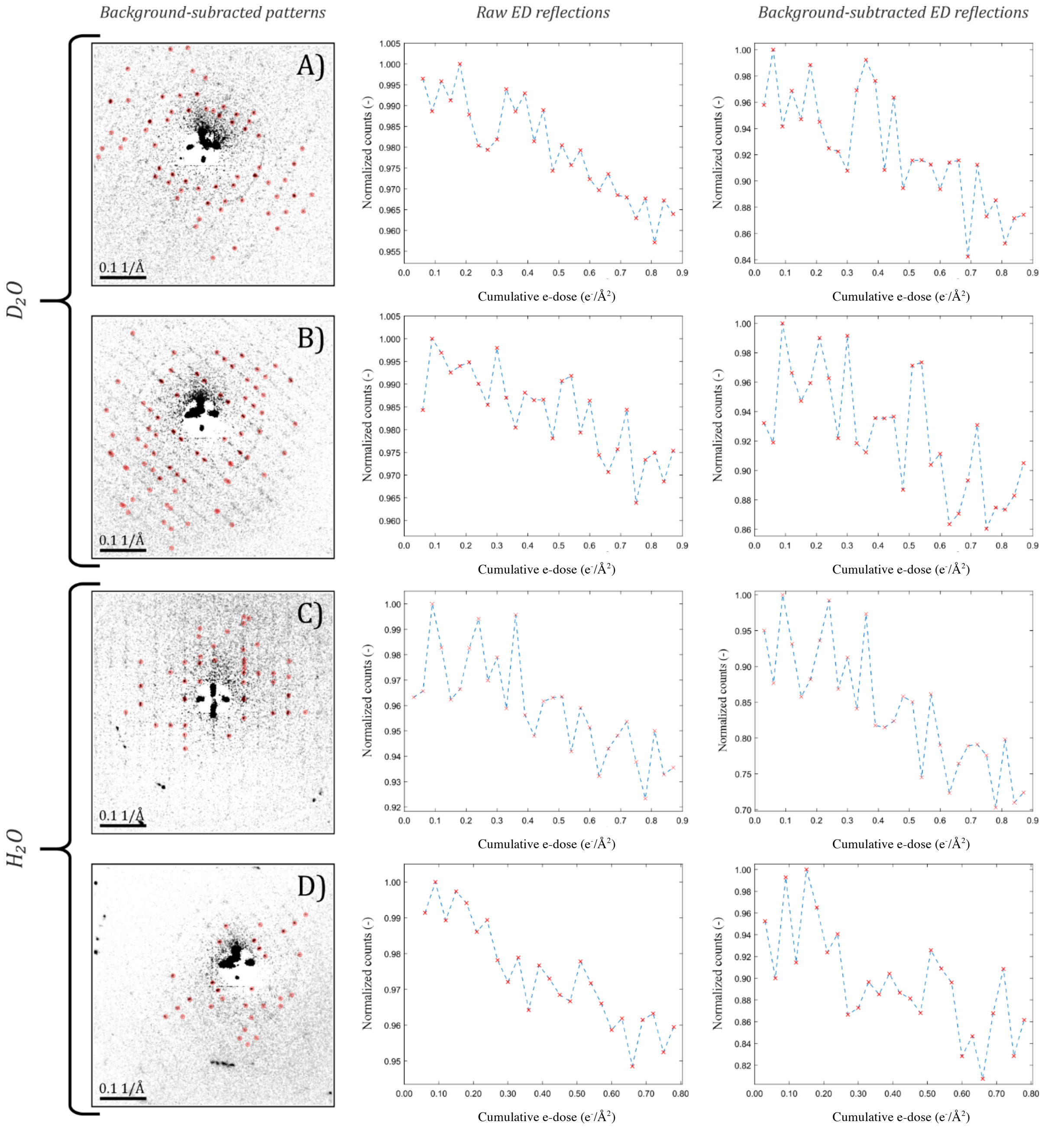

Figure S2. Background-subtracted electron diffraction (ED) patterns and plots of the decay of the reflection intensities with respect to the cumulative e-dose for four different lysozyme crystals from raw (middle) and background-subtracted (right) patterns. Top two examples are

from crystals in D_2O buffer solution and the bottom two are from crystals in H_2O buffer solution. All crystals are encapsulated in graphene liquid cells (GLCs) with 3-5 graphene layers. The red-filled circles in the ED patterns indicate the positions where reflection intensities are observed and used for the summation of the total single-crystal diffraction intensity for each consecutive integrated pattern (100 frames) of the acquired ED series (3000 frames in total; 30 seconds of total exposure time). Reflections between 12.5 Å to 3.9 Å of resolution are considered in A), 15.7 Å to 3.4 Å in B), 20 Å to 5 Å in C), and 16.5 Å to 5.2 Å in D).

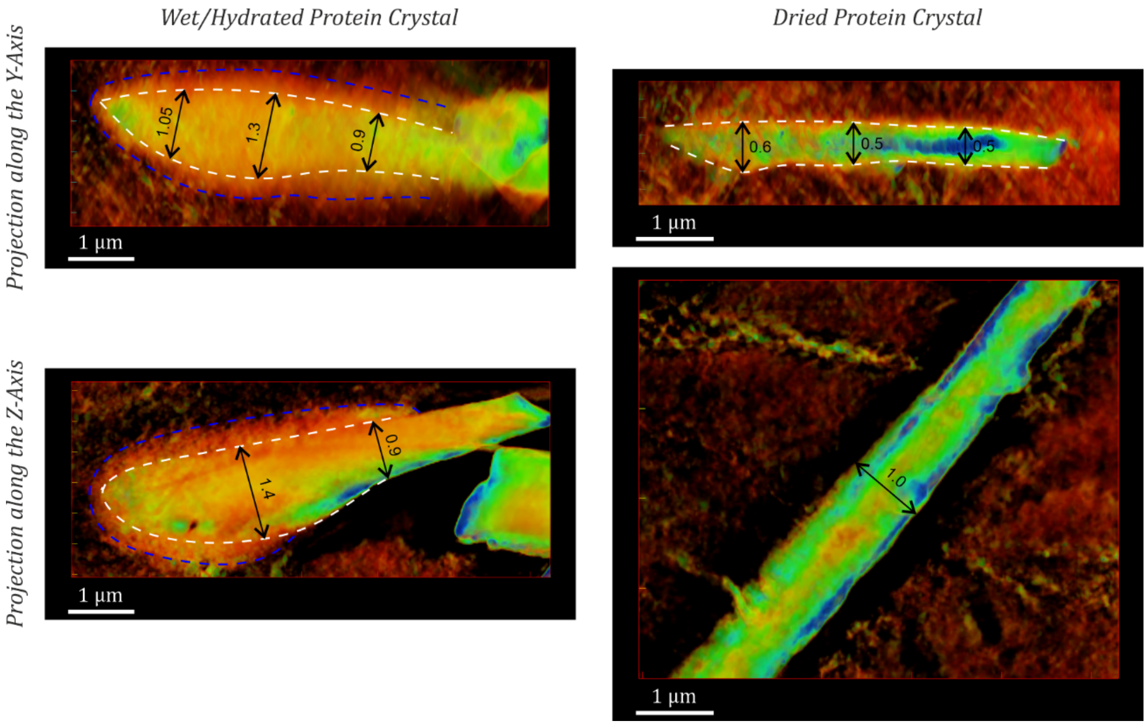

Figure S3. Projections along the y and z axes of the reconstructed volume obtained from the image tomography of the two crystals present (Figure 3 in main text). The white-dashed lines delimit the edge of the crystals, and the blue-dashed lines mark the outer limits of the liquid encapsulating the particles. The crystal dimensions are specified in micrometers with an experimental error of 0.05 μm.

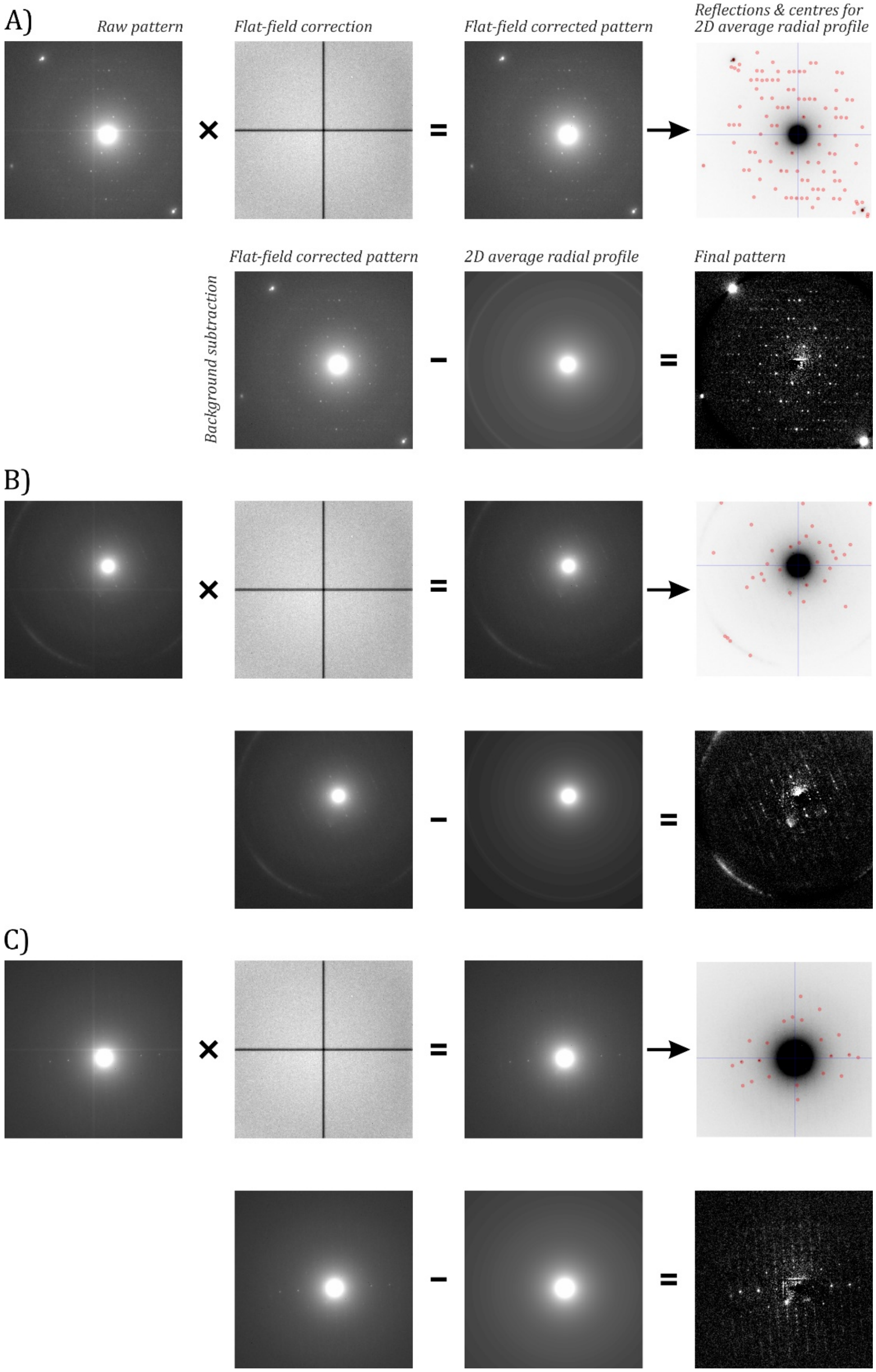
A)
Raw pattern
Flat-field correction
Flat-field corrected pattern
Reflections & centres for 2D average radial profile
Flat-field corrected pattern
2D average radial profile
Final pattern
Background subtraction
B)
C)

Figure S4. Data processing applied to the ED patterns of Figs. 4A, 4B and 4C in the main text. First, flat-field correction of the detector is applied to the raw pattern. The corrected pattern is then used to locate the center of the diffraction pattern (transmitted beam) and the observable reflections. The 2D average radial profile is obtained by considering all pixels of the pattern excluding the pixels around the reflections. Finally, the average background profile is subtracted from the flat-field corrected pattern to obtain the final ED pattern.

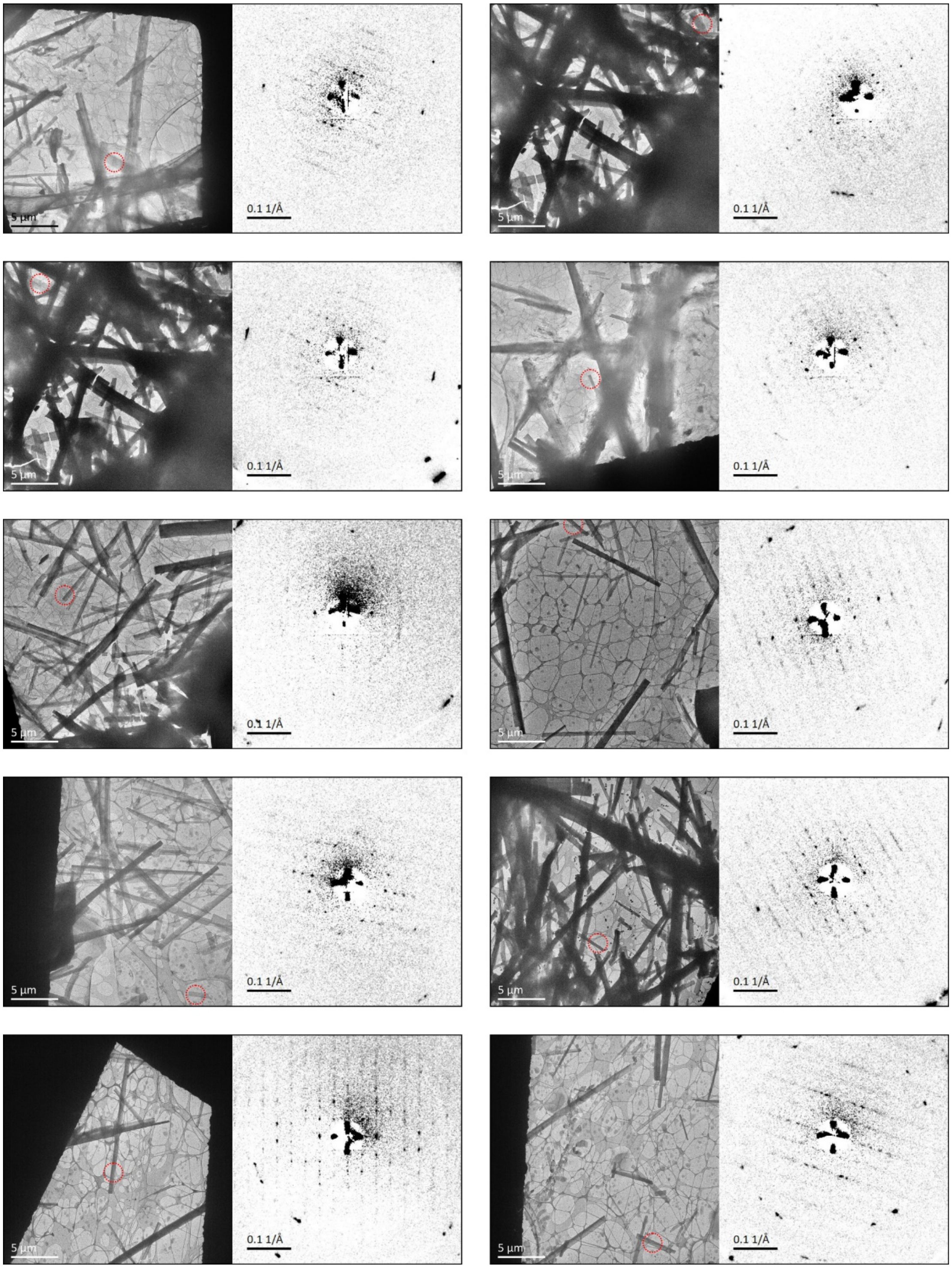

Figure S5. Representative TEM images and their associated ED patterns of lysozyme crystals in buffer solution of H_2O (cumulative e-dose of ~ 1 e/Å^2). The red dashed circles correspond to the area contributing to the ED pattern as defined by the selected area (SA) aperture.

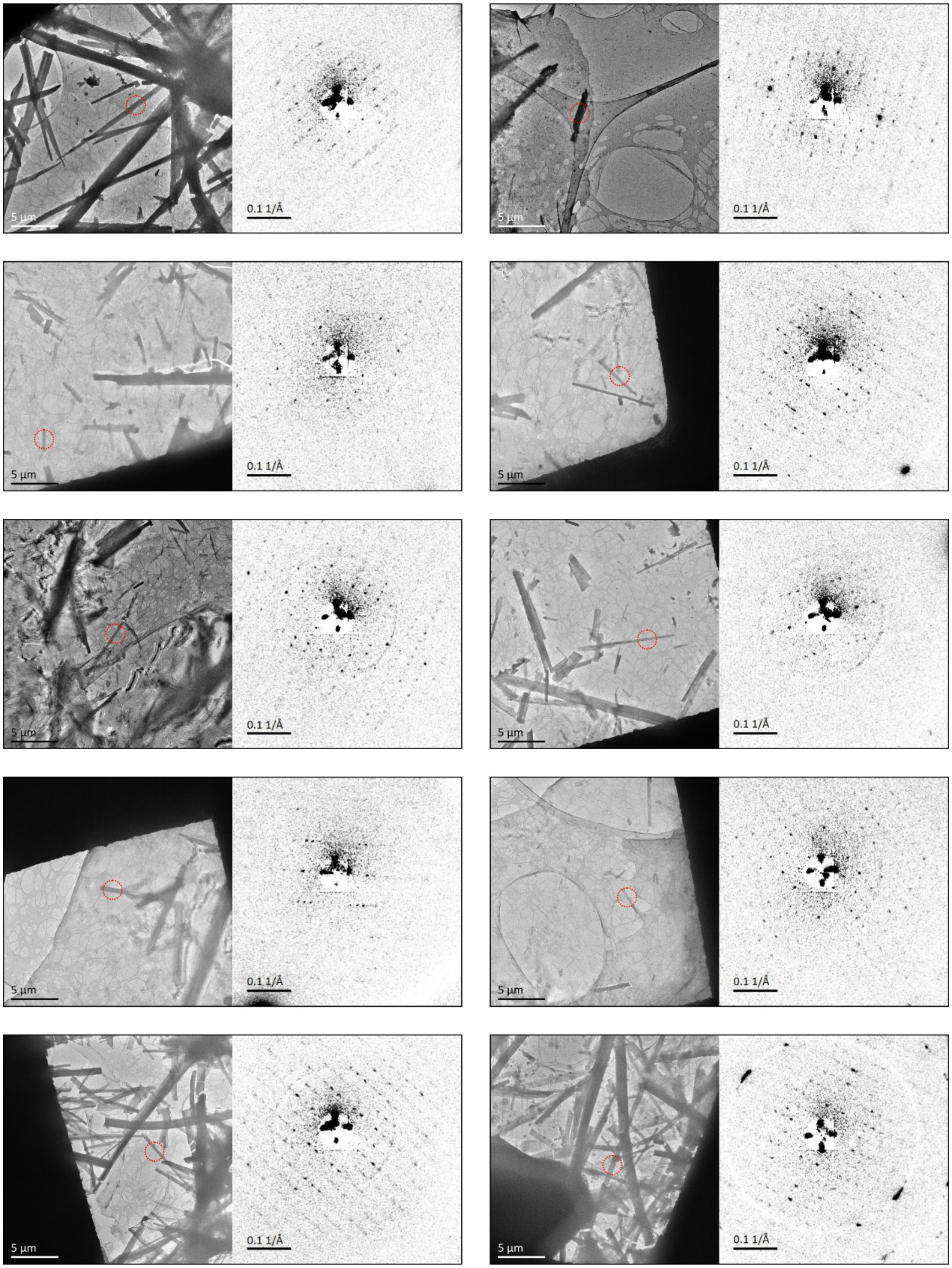

Figure S6. Representative TEM images and their associated ED patterns of lysozyme crystals in buffer solution of D_2O (cumulative e-dose of ~ 1 e/Å^2). The red dashed circles correspond to the area that contributed to the ED pattern as defined by the SA aperture.

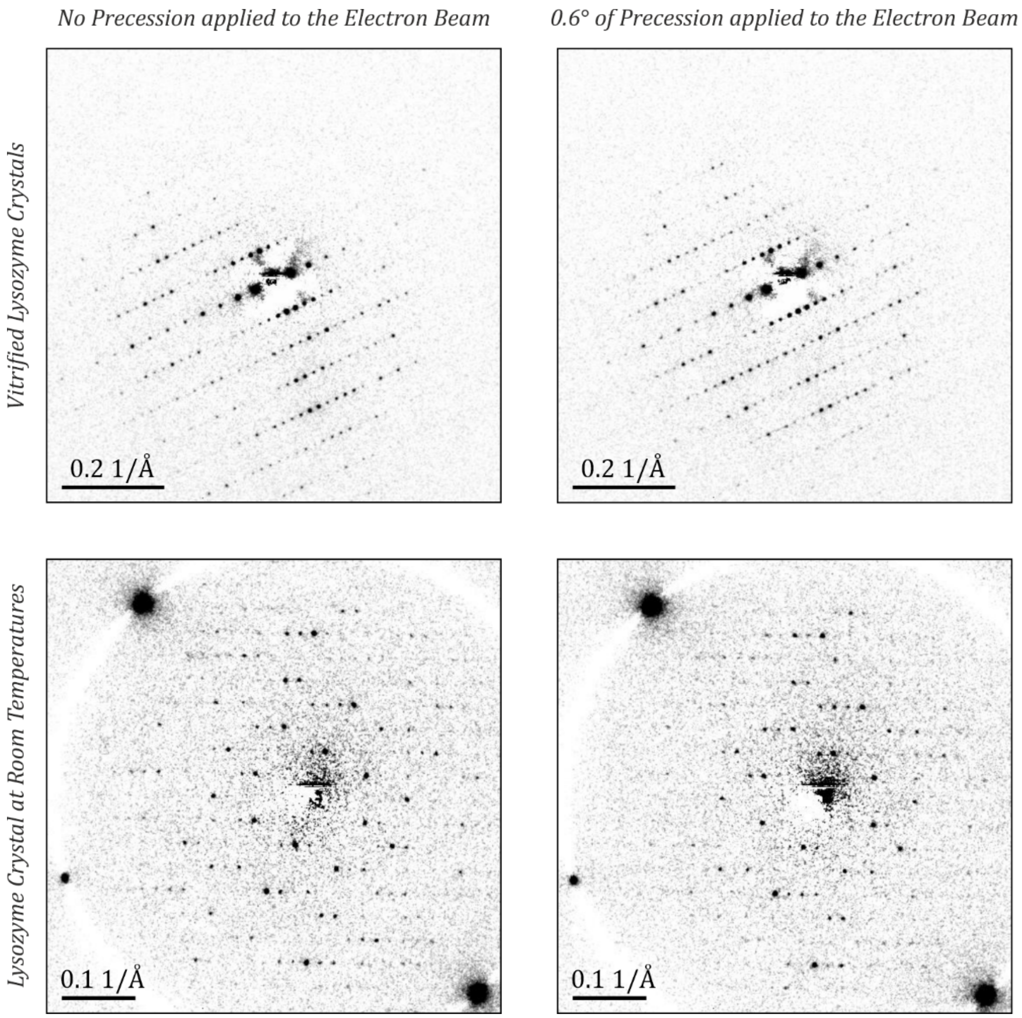

Figure S7. Diffraction patterns of lysozyme crystals acquired with and without precession (0.6°) of the electron beam. Patterns with precession have more symmetry-related reflections for both the vitrified crystal acquired at close to liquid N_2 temperature (cryoEM) and the GLC-encapsulated crystal probed at room temperature. The precession-assisted ED pattern of the room temperature crystal was acquired after the acquisition of the non-precessed pattern (after ~ 1 e^-/Å^2 of cumulated electron dose).
